# The effect of continuous electronic fetal monitoring on mode of delivery and neonatal outcome among low-risk laboring mothers at Debre Markos comprehensive specialized hospital, Northwest Ethiopia

**DOI:** 10.3389/fgwh.2024.1385343

**Published:** 2024-06-24

**Authors:** Tirusew Nigussie Kebede, Kidist Ayalew Abebe, Moges Sisay Chekol, Tebabere Moltot Kitaw, Muhabaw Shumye Mihret, Bezawit Melak Fentie, Yared Alem Sibhat, Michael Amera Tizazu, Solomon Hailemeskel Beshah, Birhan Tsegaw Taye

**Affiliations:** ^1^ Department of Midwifery, Asrat Woldeyes Health Science Campus, Debre Berhan University, Debre Berhan, Ethiopia; ^2^ Department of Clinical Midwifery, School of Midwifery, College of Medicine and Health Science, University of Gondar, Gondar, Ethiopia; ^3^ Department of General Midwifery, School of Midwifery, College of Medicine and Health Science, University of Gondar, Gondar, Ethiopia; ^4^ Department Obstetrics and Gynecology, College of Medicine and Health Science, University of Gondar, Gondar, Ethiopia; ^5^ Department Public Health, Asrat Woldeyes Health Science Campus, Debre Berhan University, Debre Berhan, Ethiopia

**Keywords:** electronic fetal monitoring, caesarean delivery, intermittent auscultation, labor, asphyxia, instrumental delivery

## Abstract

**Background:**

Electronic fetal heart rate monitoring (EFM) has been widely used in obstetric practice for over 40 years to improve perinatal outcomes. Its popularity is growing in Ethiopia and other sub-Saharan African countries to reduce high perinatal morbidity and mortality rates. However, its impact on delivery mode and perinatal outcomes in low-risk pregnancies remains controversial. This study aimed to assess the effect of continuous EFM on delivery mode and neonatal outcomes among low-risk laboring mothers at Debre Markos Comprehensive Specialized Hospital, Northwest Ethiopia.

**Methods:**

A prospective follow-up study was conducted from November 20, 2023, to January 10, 2024. All low-risk laboring mothers meeting the inclusion criteria were included. Data were collected via pretested structured questionnaires and observation, then analyzed using Epi-data 4.6 and SPSS. The incidences of cesarean delivery and continuous EFM were compared using the chi-squared test and Fisher's exact test.

**Results:**

The study found higher rates of instrumental-assisted vaginal delivery (7% vs. 2.4%) and cesarean sections (16% vs. 2%) due to unsettling fetal heart rate patterns in the continuous EFM group compared to the intermittent auscultation group. However, there were no differences in immediate neonatal outcomes between the groups.

**Conclusion:**

When compared to intermittent auscultation with a Pinard fetoscope, the routine use of continuous EFM among low-risk laboring mothers was associated with an increased risk of cesarean sections and instrumental vaginal deliveries, without significantly improving immediate newborn outcomes. However, it is important to note that our study faced significant logistical constraints due to the limited availability of EFM devices, which influenced our ability to use EFM comprehensively. Given these limitations, we recommend avoiding the routine use of continuous EFM for low-risk laboring mothers to help reduce the rising number of operative deliveries, particularly cesarean sections. Our findings should be interpreted with caution, and further research with adequate resources is needed to draw definitive conclusions.

## Introduction

In 2020, 2.4 million infants worldwide lost their lives in the first month of birth. Every day, almost 6,700 newborns lose their lives. About 1 million babies died within the first 24 h in 2019, with birth asphyxia being one of the main causes. The majority of neonatal deaths (75%) occur within the first week of life (1, 2). The main objective of obstetric care is the birth of healthy babies, to lower the risks of unfavorable outcomes for both the mother and the fetus, a number of technologies, including EFM, have been developed (3). In an effort to detect fetal oxygen deprivation in its early stages and maybe enable medical personnel to intervene before hypoxic brain impairment occurs, heart rate monitoring is used during delivery (4).

Continuous EFM is the ongoing monitoring of fluctuations of the fetal heart rate (FHR) in response to maternal contractions and is considered standard practice during active labor (5, 6). It was developed in the 1920s (4). Since the 1970s, it has been extensively used in labor all over the world (7). Continuous EFM is designed to detect early fetal hypoxia and thereby decrease neonatal morbidity and mortality compared with intermittent auscultation (8). However, EFM has a 99% false-positive rate of fetal hypoxia (4). Continuous EFM is important to prevent neonatal seizure and fetal asphyxia and its complication (3, 4, 9, 10).

Although it is appropriate to use for high-risk labor, the widespread use of continuous EFM has increased the rates of instrumental and cesarean deliveries without improving infant outcomes (4). Continuous EFM during labor was associated with an 81% higher chance of primary cesarean section in both high-risk and low-risk pregnancies, according to a scoping review report (11). Further research revealed that the use of continuous EFM raised the percentage of cesarean deliveries to 33% without correspondingly lowering the incidence of cerebral palsy (12). According to a retrospective investigation over 20 years, there was a 14% and 24% increase in risk of primary cesarean delivery and instrumental vaginal births, respectively, associated with EFM (13). EFM increases the risk of cesarean delivery by 66% and the rate of surgical vaginal delivery by 16%, according to a systematic review of randomized control trials (8). The rate of cesarean deliveries has increased from less than 5% in the 1970s to more than 30% at now due to the frequent use of EFM (14).

The rates of acidemia, cesarean deliveries, and perinatal deaths did not differ between organized intermittent auscultation and continuous EFM, according to a sizable before-and-after research (15). When used during low-risk labor, structured intermittent auscultation can reduce the number of operative deliveries while producing newborn results comparable to continuous EFM. It is an underutilized type of fetal monitoring, especially in developed countries and higher-level health institutions. However, systematic intermittent auscultation is still challenging to apply because of shortages in midwives' staffing and physician monitoring (16). The evidence on EFM, delivery mode, and newborn outcome is inconsistent. In Ethiopian higher health institution including the study area continuous EFM is widely utilized. However, its relation on mode of delivery and neonatal outcome had not been studied. Thus, the purpose of this study is to evaluate these factors at the Debre Markos comprehensive specialty hospital.

## Method and materials

### Study design, period and setting

From November 20, 2023, to January 10, 2024, a prospective follow-up study was carried out at Debre Markos Comprehensive Specialized Hospital in the East Gojjam Zone of the Amhara regional state in Northwest Ethiopia. The hospital is found 299 km northwest of Addis Ababa, a capital city of Ethiopia and 268 km from Bahir Dar, a capital of Amhara regional state. Debre Markos comprehensive specialized hospital is the only referral hospital in Gojjam next to Tibebeghion and Felege Hiwot hospital at Bahir Dar. It gives a routine and comprehensive health service for an estimated population of more than five million found in the zone and nearby border areas. The hospital averagely provides around 7,000 delivery service annually. The hospital had six labors follow up bed and four second stage delivery bed. It had four functional EFM which is cardiotocography (CTG) and five pinard fetoscope which is used for fetal heart beat monitoring in the laboring process.

### Populations

All laboring women who were followed and attended at Debre Markos comprehensive specialized hospital Amhara, Northwest Ethiopia, 2023.

### Eligibility criteria

The study included all full-term low-risk mothers who were admitted with a cervical dilatation of less than 8 cm and who at least monitored their laboring process beginning with the early active first stage of labor. However, the study excluded participants who had oligohydramnios, intrauterine growth restriction, grade II or higher meconium-stained amniotic fluid during latent or early active first stage of labor, previous history of caesarean delivery including myomectomy, induction, as well as obstetrical and medical conditions that were strongly linked to NRFHRP and could have an impact on the mode of delivery.

### Sample size determination, sampling procedure and selection of eligible population

During the study period, all laboring mothers who met the eligibility criteria were included in the sample size, and their complete information was collected. High-risk pregnancies and labor conditions were prioritized for continuous electronic fetal monitoring (EFM). However, due to a limited number of healthcare providers, low-risk laboring women were also monitored using the available EFM. On average, two high-risk women were continuously monitored with EFM at this hospital during the study period. All eligible laboring women were included in the study, regardless of whether they were monitored with continuous EFM or intermittent auscultation (IA). For the purposes of the study, low-risk women monitored with continuous EFM were categorized as the EFM-exposed group, while those monitored with IA were categorized as the control group.

### Variable of the study

Outcome variable: Mode of delivery and immediate neonatal outcome.

Independent variable: Continuous electronic fetal monitoring.

### Operational definition

Full-term delivery: Defined as a birth occurring between37 and 41 completed weeks gestation (11).

Low-risk pregnancy: Defined as full-term, singleton (11). Vertex presentation, no meconium staining, intrapartum bleeding, abnormal fetal heart rate results upon initial admission; no increased risk of developing fetal acidemia during labor (e.g., congenital anomalies, intrauterine growth restriction, oligohydramnios or polyhydramnios and Reduced fetal movements in the last 24 h reported by the woman); no maternal condition that may affect fetal well-being (e.g., prior uterine scar including cesarean scar and myomectomy, diabetes, hypertensive disease, thyroid disorder, obesity); and no requirement for oxytocin induction or augmentation of labor, premature rapture of labor, chorioamnionitis, malpresentation (17, 18).

Continuous EFM: Defined as electronic monitoring of the fetal heart rate using an external ultrasound transducer continuously through the laboring process (5).

Intermittent auscultation: Is the technique used to listen to the fetal heart rate (FHR) for short periods of time without a display of the resulting pattern. Whether it is used for intrapartum fetal monitoring in low-risk women or for all cases in settings where there are no available alternatives (19), During this process, FHR is assessed immediately after a contraction for 1 min in the latent first stage of labor, every hour, in the active first stage, it is monitored every 30 min, and in the second stage of labor, it is checked every 15 min (20). Caesarean delivery: Is the delivery of the fetus and placenta through abdominal and uterine incision after fetal viability.

Operative vaginal delivery: Refers to a delivery in which the operator uses forceps or a vacuum device to extract the fetus from the vagina, with or without the assistance of maternal pushing (21).

Immediate neonatal outcome: Refers to adverse outcomes (stillbirth, low 1st and 5th Apgar score, neonatal admission to NICU with diagnosis of neonatal asphyxia) until the women and her neonate discharged from the hospital.

### Data collection tool and procedure

To gather relevant data for the study, a combination of structured interviews and document review methods were utilized. The researchers developed a set of structured interview questions based on a review of relevant literature (4, 6, 9, 11–13). This questionnaire encompassed socio-demographic characteristics of the laboring mother, as well as her obstetrics and medical history. Certain variables were extracted from the medical records.

In terms of data collection procedures, laboring mothers who arrived at the hospital first were monitored using EFM or CTG, while the remaining parturient women were monitored using a pinard fetoscope. The frequency of monitoring varied, with intervals of 30 min during the active first stage of labor and 15 min during the second stage. Before admitting of the laboring women, the baseline of FHR was determined with pinard fetoscope at the triage room. These monitoring protocols were standard practice at the hospital.

The data were collected by a team of six Bachelor of Science Midwives. Three of them collected data from women undergoing continuous EFM, while the other three collected data from parturient women monitored through intermittent auscultation. The data collection process was supervised by a Master of Science specialist in clinical Midwifery and a senior obstetrician and gynecologist.

### Data quality control

The questionnaire was initially developed in English and then translated into Amharic, a commonly spoken language. Afterwards, it was translated back into English to ensure consistency. To ensure the questionnaire's clarity, understandability, and simplicity, a pretest was conducted on 5% of the estimated sample population at Feleghiwot Comprehensive Specialized Hospital. The data collectors and supervisors received training on proper data collection techniques and supervision. The principal investigator closely oversaw the entire data collection process and worked closely with the supervisors. Data were collected both during the day and at night by the data collectors. Each day, the principal investigator and supervisors checked the data for completeness.

### Data processing and analysis

The collected data underwent a series of steps for quality control and analysis. First, the data were checked for accuracy, coded, and entered into Epi-data version 4.2. Afterward, the data were exported to SPSS version 26 for further analysis. Descriptive statistics and cross tabulation techniques were employed to clean the data, ensure completeness, identify missing values, and detect any unusual or outlier data. To compare the rates of cesarean delivery and continuous EFM, statistical tests such as the chi-squared test and Fisher's exact test were conducted. A significance level of *p* < 0.05 was used to determine statistical significance. Finally, the results were presented using appropriate tables, charts, and accompanying text to effectively communicate the findings of the study.

### Ethical consideration

Ethical clearance was obtained from University of Gondar, college of medicine and health science school of Midwifery under the delegation of the university institutional ethical review board (Ref; MIDW/38/2023). And formal letter of study approval was obtained from Debre Markos comprehensive specialized hospital administrators. Finally written and oral consent was taken from each individual study participants after informed about the important of the study, how the Study carried out, the potential risks and benefits and all about the study. Participants address, names and signs couldn't be included in order to keep participants confidentiality.

## Result

### Socio-demographic characteristics

A total of 594 laboring mothers in Debre Markos comprehensive specialized hospital were enrolled in this study during the study period with 99% of response rate. The median age of the study participants was 27 with interquartile range of six. Most of the study participants 336 (57.1%) were in the age group of 25–34 year. Almost all of the study participants 582 (99%) was the follower of orthodox religion. Majority of the study participants 581 (98.8%) were married (Table 1).

**Table 1:** Socio demographic characteristics of study participant at Debre Markos comprehensive specialized hospital, 2024 (*n* = 588).

Variables	Category	Electronic fetal monitoring [CTG] (%)	Intermittent fetal monitoring [pinard fetoscope] (%)
Age	15–24	99 (35.6%)	83 (26.8%)
	25–34	153 (55%)	183 (59%)
	35–49	26 (9.4%)	44 (14.2%)
Religion	Orthodox	274 (98.6%)	308 (99.4%)
	Muslim	2 (0.7%)	1 (0.3%)
	Protestant	2 (0.7%)	1 (0.3%)
Marital status	Single	0	4 (1.3%)
	Cohabited	1 (0.4%)	1 (0.3%)
	Married	277 (99.6%)	304 (98.1%)
	Divorced	0	1 (0.3%)
Occupation	House wife	107 (38.5%)	114 (36.8%)
	Government employee	68 (24.5%)	60 (19.4%)
	Private business	32 (11.5%)	43 (13.9%)
	Merchant	11 (4%)	12 (3.9%)
	Farmer	59 (21.2%)	81 (26.1%)
	Daily laborer	1 (0.4%)	0
Educational status	Can't read & write	40 (14.4%)	47 (15.2%)
	Read & write	21 (7.6%)	35 (11.3%)
	1–8 grade	62 (22.3%)	70 (22.6%)
	9–12 grade	78 (28.1%)	74 (23.9%)
	Collage and above	77 (27.7%)	84 (27.1%)
Residence	Urban	202 (72.7%)	198(63.9%)
	Rural	76(27.3%)	112(36.1%)

### Pregnancy related history of the study participant

A majority of the study participants, comprising 417 (70.9%), were classified as multigravida. Similarly, 417 (70.9%) of the pregnancies reported were intentional and supported, reflecting a high level of planning and desired pregnancies among the participants. It was also observed that a large proportion of the study participants, specifically 559 (93.2%), had attended at least one antenatal care (ANC) visit during their current pregnancy (Table 2).

**Table 2:** Pregnancy related history of laboring mothers at Debre Markos comprehensive specialized hospital 2024 (*n* = 588).

Variables	Category	Electronic fetal monitoring [CTG] (%)	Intermittent fetal monitoring [fetoscope] (%)
Gravidity	Primigravida	96 (34.5%)	75 (24.2%)
	Multigravida	182 (65.5%)	235 (75.8%)
Parity	Nulliparous	106 (38.1%)	85 (27.4%)
	Multipara	172 (61.9%)	225 (72.6%)
Antenatal care contact	At least one	257 (92.4)	292 (94.2%)
	No	21 (7.6%)	18 (5.8%)
Type of pregnancy	Wanted	265 (95.3%)	298 (96.1%)
	Unwanted	13 (4.7%)	12 (3.9%)
Was the pregnancy supported	Supported	271 (97.5%)	302 (97.4%)
	Unsupported	7(2.5%)	8(2.6%)

### Continuous electronic fetal monitoring and mode of delivery

In the study area, the incidence of caesarean delivery among low-risk laboring mothers was 24.3%. Among these caesarean deliveries, 18% were performed due to a non-reassuring fetal heart rate pattern (NRFHRP). Notably, the incidence of caesarean delivery resulting from NRFHRP was higher in cases where continuous EFM was utilized (16%), compared to cases where intermittent pinard fetoscope auscultation was used (2%). The remaining 37 (6.3%) were delivered with CD with indication other than NRFHRP.

**Figure 1:**
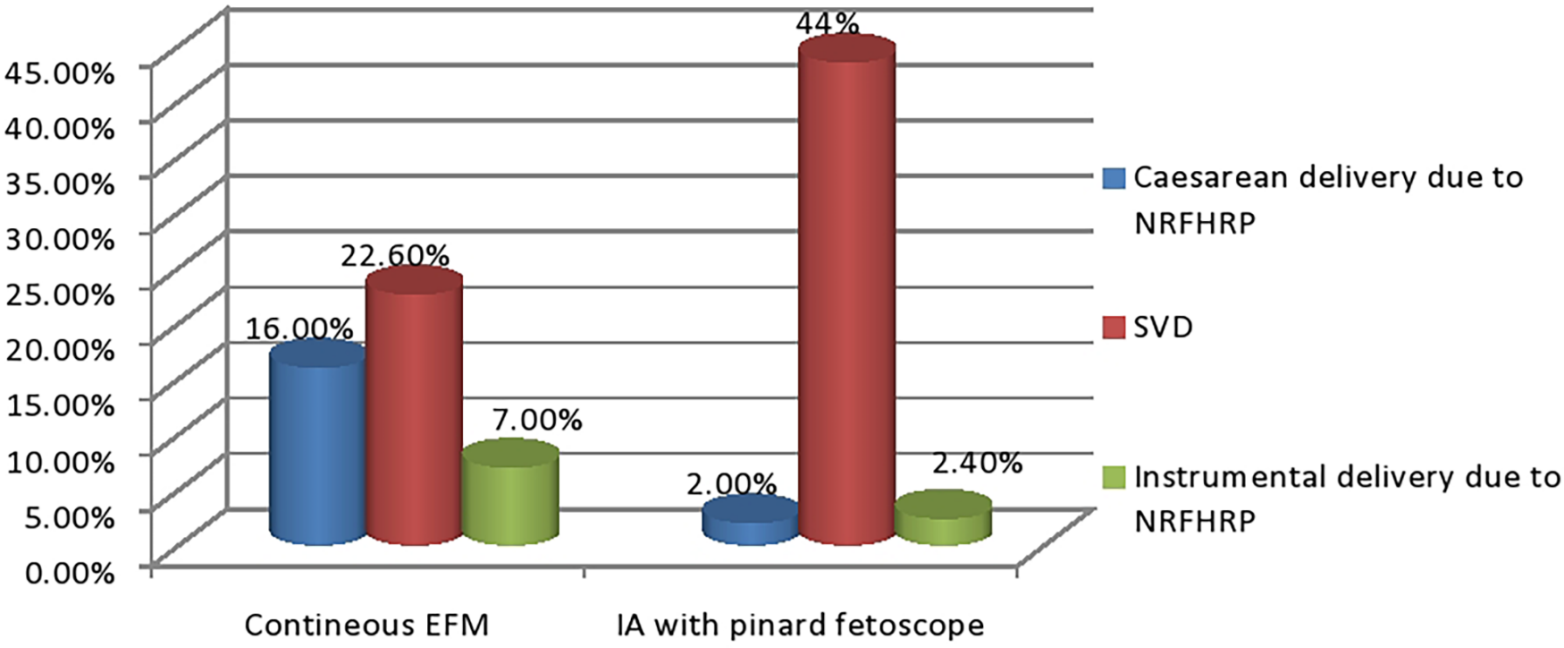
The relationship between methods of fetal monitoring and mode of delivery among low-risk laboring mothers at Debre Markos comprehensive specialized hospital, north west Ethiopia 2024.

The burden of instrumental delivery, specifically vacuum delivery, was 9.4%. It is worth mentioning that forceps delivery was not permitted for every healthcare professional in the hospital due to previous instances of maternal and fetal complications associated with its use. Approximately 7% of instrumental deliveries were conducted following continuous EFM (Figure 1).

### Continuous electronic fetal monitoring and immediate neonatal outcomes

When considering immediate neonatal outcomes, such as stillbirth, 1st and 5th minute Apgar scores, and neonatal admission to the intensive care unit (NICU) due to neonatal asphyxia, no significant difference was observed between continuous EFM and intermittent auscultation (IA) with a pinard fetoscope. Among neonates monitored using continuous EFM, 4% had a 5th minute Apgar score of less than seven. However, this difference was not statistically significant compared to the 5.2% of neonates monitored using IA with a pinard fetoscope. For both groups stillbirth and neonatal death was not recorded or occurred (Table 3).

**Table 3:** Continuous EFM and immediate neonatal outcomes among low-risk laboring mothers at Debre Markos comprehensive specialized hospital 2024 (*n* = 588).

Immediate neonatal outcomes	Continuous EFM (%)	IA with pinard fetoscope (%)	Significance test		
			Pearson *X*^2^	*P* value	Risk (95% CI)
1st minute Apgar score <7	25 (9%)	31 (10%)	0.17	0.67	0.89 (0.54, 1.48)
1st minute Apgar score ≥7	253 (91%)	279 (90)	–	–	–
5th minute Apgar score <7	11 (4%)	16 (5.2%)	0.48	0.48	0.76 (0.36, 1.62)
5th minute Apgar score ≥7	267 (96%)	294 (94.8%)	–	–	–
Neonatal admission to NICU with a diagnosis of neonatal asphyxia	7 (2.5%)	10 (3.2%)	0.262	0.61	0.78 (0.31, 2.02)

## Discussion

To evaluate the well-being of the fetus during labor, monitoring the fetal heart rate (FHR) is a common practice. This can be done through intermittent auscultation, continuous EFM, or CTG. Both monitoring techniques are applicable to the study area. The findings of the study highlight the significance of FHR monitoring methods in influencing the mode of delivery and immediate outcomes for the newborn. Thus, the findings of the study revealed several noteworthy points. Firstly, the overall incidence of caesarean delivery among low-risk laboring mothers was relatively high at 24.3%. Among these caesarean deliveries, 18% were attributed NRFHRP.

Interestingly, the study found a significant difference in the incidence of caesarean delivery due to NRFHRP between continuous EFM and IA with a pinard fetoscope. The incidence was considerably higher (16%) in cases where continuous EFM was utilized compared to cases where IA with a pinard fetoscope was used (2%). This suggests that continuous EFM may lead to a higher rate of caesarean deliveries *in situ*ations involving NRFHRP. Furthermore, the burden of instrumental delivery, primarily vacuum delivery, was observed to be 9.4% among the low-risk laboring mothers. It is worth noting that forceps delivery was not routinely practiced due to previous maternal and fetal complications associated with its use. Among instrumental deliveries, approximately 7% were performed following continuous EFM.

In terms of immediate neonatal outcomes, including stillbirth, Apgar scores at 1st and 5th minutes, and neonatal admission to the NICU due to neonatal asphyxia, no significant difference was found between continuous EFM and IA with a pinard fetoscope. The percentage of neonates with a 5th minute Apgar score below seven was 4% in the continuous EFM group, which did not significantly differ from the 5.2% in the IA with pinard fetoscope group.

These findings suggest that continuous EFM may be associated with a higher incidence of caesarean delivery in cases of NRFHRP among low-risk laboring mothers. However, there was no significant difference in neonatal outcomes between continuous EFM and IA with a pinard fetoscope. These results highlight the need for careful consideration when deciding on the appropriate monitoring method during labor, balancing the potential benefits and risks associated with continuous EFM. Further research is warranted to explore the long-term outcomes and potential implications of continuous EFM on both maternal and neonatal health in low-risk laboring mothers.

The finding of this study are in line with scoping review since 1996 showed that continuous EFM increase the risk of caesarean delivery by 80% and operative instrumental vaginal delivery by 23% (11) in low risk pregnancies. Another study also concluded that continues use of EFM was increased the incidence of caesarean delivery due to NRFHRP by 33% without improving the rate of neonatal asphyxia compared to IA in low-risk pregnancy; which is agreed with the finding of this study (12). A secondary data analysis in United States also reported that continuous EFM increased the incidence of primary caesarean delivery by 10%–40% and instrumental assisted vaginal delivery by 14%–24% without any difference in intrapartum stillbirth and neonatal mortalities and morbidity; which is in line with the finding of this study (13). A Cochrane review of randomized control trials reported that EFM increases the incidence of cesarean section by 66% and the incidence of operative vaginal delivery by 16% with no effect on the rates of cerebral palsy or neonatal mortality; this is supportive evidence of the current study (8). The findings of a Cochrane review report also corroborate this observation, indicating that continuous electronic fetal monitoring (EFM) significantly increases the rate of operative deliveries across various risk categories, including low-risk, high-risk, and preterm births. Importantly, there was no notable difference in neonatal outcomes, yet there was a reduction in the rate of neonatal seizures (5). Another Cochrane review reinforces our discovery, indicating that continuous fetal monitoring with CTG increases the likelihood of cesarean delivery by 20% among low-risk labor cases. The review suggests that abstaining from FHB monitoring with CTG in low-risk patients is the optimal approach (22).

The indication for caesarean delivery in this study was fetal heart rate (FHR) deceleration. These decelerations during labor are believed to be mediated by the peripheral chemoreflex and are associated with temporary but acute episodes of asphyxia. It is common for hypoxic episodes to occur with each uterine contraction, and a healthy fetus demonstrates a remarkable ability to adapt to these repetitive episodes of asphyxia (23–25).

Even after numerous brief decelerations during uncomplicated labor, the healthy fetus is usually not at risk of hypotension and damage (26). FHR decelerations typically result from compression of the fetal head or cord, whereas decelerations in EFM calibrations are typically linked to unthreading of the peripheral chemo-reflex (26, 27). One of the most basic ones would be the lack of clarity surrounding the potential applications of EFM, the imprecision of its measurements combined with a significant degree of variability in its interpretation, and the wide range of applications to intrapartum management (3, 24). Continuous EFM has associated with high false positive rate which may leads to unnecessary interventions in low risk labor (28); thus, although its usage is justified in high-risk labor, the widespread use of continuous EFM in low risk labor has raised the rates of caesarean and operative vaginal deliveries without improving newborn outcomes (16, 29).

In addition, it is also possible that the low-Fowler's positioning commonly used to promote optimal EFM output may reduce pressure of the fetal head on the cervix, leading to an increased need for oxytocin augmentation, which also has a demonstrated association with NRFHRP and then increased rates of cesarean birth (11). Furthermore, most of the study participants on continuous EFM are on supine position. This might lead to orthostatic hypotension due to compression of the inferior vena cava which decreases the venous return and it causes insufficient utero placental perfusion and results NRFHRP. One potential reason for this elevated risk might be the reduced mobility experienced by women while connected to electronic fetal monitors. This decreased mobility has the potential to trigger a chain of additional interventions that can impact the duration of labor, ultimately contributing to prolonged labors. Moreover, a woman's ability to move freely during labor is associated with decreased pain, improved maternal-fetal circulation, and enhanced quality of uterine contractions. However, these benefits are often restricted for women undergoing continuous electronic fetal monitoring (EFM), which may significantly increase the risk of operative deliveries (13, 30). In the study area, none of the healthcare providers are specifically trained in the interpretation and reading of fetal heartbeats (FHB) on continuous EFM or CTG. This lack of training may also contribute to the observed challenges.

The combination of the aforementioned factors significantly contributes to the rising incidence of caesarean deliveries due to NRFHRP in low-risk pregnancies that are monitored using continuous EFM, all without demonstrating improvement in neonatal outcomes. Consequently, both the American College of Obstetricians and Gynecologists (ACOG) and the World Health Organization (WHO) have recommended minimizing the use of continuous EFM in low-risk pregnancies as a means of controlling the escalating rates of caesarean deliveries worldwide (31, 32).

### Strength and limitation of the study

This study, conducted in a real-world setting, offers valuable insights into the practical challenges and decision-making processes in resource-limited environments, particularly regarding the use of continuous electronic fetal monitoring (EFM) for low-risk laboring mothers. However, the study has significant limitations, including the limited availability of EFM devices, which affected the frequency and context of their use, and the inability to draw more definitive conclusions. Furthermore, as a single-center study with potential selection bias, our findings may not be universally applicable.

## Conclusion

In our study, the limited availability of EFM devices presented a significant logistical challenge, affecting both the frequency and context of EFM usage. Although our findings suggest that operative deliveries (caesarean sections and instrumental deliveries) among low-risk laboring women were more frequent in the EFM group compared to the intermittent auscultation (IA) group, immediate neonatal outcomes without continuous EFM were not significantly different from those with EFM. However, this observation should be interpreted with caution, as our study lacks the statistical power to draw definitive scientific conclusions about the non-necessity of EFM. Our experience highlights the need for a pragmatic approach in resource-limited settings, where optimal use of available resources based on scientific evidence is essential. We recommend conducting further research to address the limitations of this study, particularly in low resource settings.

## Data Availability

The raw data supporting the conclusions of this article will be made available by the authors, without undue reservation.
